# Chloroplast and mitochondrial genetic variation of larches at the Siberian tundra-taiga ecotone revealed by *de novo* assembly

**DOI:** 10.1371/journal.pone.0216966

**Published:** 2019-07-10

**Authors:** Heike H. Zimmermann, Lars Harms, Laura S. Epp, Nick Mewes, Nadine Bernhardt, Stefan Kruse, Kathleen R. Stoof-Leichsenring, Luidmila A. Pestryakova, Mareike Wieczorek, Daronja Trense, Ulrike Herzschuh

**Affiliations:** 1 Polar Terrestrial Environmental Systems Research Group, Alfred Wegener Institute Helmholtz Centre for Polar and Marine Research, Potsdam, Germany; 2 Institute of Biochemistry and Biology, University of Potsdam, Potsdam, Germany; 3 Scientific Computing, Alfred Wegener Institute Helmholtz Centre for Polar and Marine Research, Bremerhaven, Germany; 4 Department of Biology, University of Konstanz, Konstanz, Germany; 5 Institute of Natural Sciences, North-Eastern Federal University of Yakutsk, Yakutsk, Russia; 6 Institute for Integrated Natural Sciences, Biology, Koblenz-Landau University, Koblenz, Germany; 7 Institute of Environmental Sciences and Geography, University of Potsdam, Potsdam, Germany; National Cheng Kung University, TAIWAN

## Abstract

*Larix* populations at the tundra-taiga ecotone in northern Siberia are highly under-represented in population genetic studies, possibly due to the remoteness of these regions that can only be accessed at extraordinary expense. The genetic signatures of populations in these boundary regions are therefore largely unknown. We aim to generate organelle reference genomes for the detection of single nucleotide polymorphisms (SNPs) that can be used for paleogenetic studies. We present 19 complete chloroplast genomes and mitochondrial genomic sequences of larches from the southern lowlands of the Taymyr Peninsula (northernmost range of *Larix gmelinii* (Rupr.) Kuzen.), the lower Omoloy River, and the lower Kolyma River (both in the range of *Larix cajanderi* Mayr). The genomic data reveal 84 chloroplast SNPs and 213 putatively mitochondrial SNPs. Parsimony-based chloroplast haplotype networks show no spatial structure of individuals from different geographic origins, while the mitochondrial haplotype network shows at least a slight spatial structure with haplotypes from the Omoloy and Kolyma populations being more closely related to each other than to most of the haplotypes from the Taymyr populations. Whole genome alignments with publicly available complete chloroplast genomes of different *Larix* species show that among official plant barcodes only the *rcbL* gene contains sufficient polymorphisms, but has to be sequenced completely to distinguish the different provenances. We provide 8 novel mitochondrial SNPs that are putatively diagnostic for the separation of *L*. *gmelinii* and *L*. *cajanderi*, while 4 chloroplast SNPs have the potential to distinguish the *L*. *gmelinii*/*L*. *cajanderi* group from other *Larix* species. Our organelle references can be used for a targeted primer and probe design allowing the generation of short amplicons. This is particularly important with regard to future investigations of, for example, the biogeographic history of *Larix* by screening ancient sedimentary DNA of *Larix*.

## Introduction

Deciduous larch (*Larix* Mill.) forests cover a vast area of about 263.2 million ha in the Russian Federation [1,2], where they form the light taiga as the only representative with tree growth form. Despite the relevance of larch as an ecological key species, the long-term history of larch forests is still poorly investigated. Climate warming in northern Siberia is expected to lead to northward expansions of larches into areas currently covered by tundra [3–5]. However, population responses to climate warming, such as recruitment patterns, are still unclear.

The Siberian treeline is a climatically driven ecotone, in which light taiga gradually opens up and changes into tundra [1]. This ecotone is located approximately where the mean July temperature lies between 10°C and 12.5°C [5]. It is characterized by a short growing season and strong seasonality [6]. In central and eastern Siberia, the treeline is formed by *Larix gmelinii* (Rupr.) Kuzen. from ~90° to ~120°E and *Larix cajanderi* Mayr from ~120° to ~160°E° [1]. The two species are closely related, and *L*. *cajanderi* was estimated to have diverged from *L*. *gmelinii* approximately during the Pliocene [7].

Macrofossil and pollen data indicate past climate-induced range contractions of *Larix* stands in northern Siberia during glacial periods and expansions during interglacial and interstadial periods [8,9]. Additionally, past biogeography, disturbances (e.g. wildfires, herbivory), and abiotic (e.g. permafrost distribution and seasonal thaw-depth) and biotic interactions (e.g. competition, hybridization) have shaped the modern larch stands [10]. However, modern-analog reconstructions of past environments are difficult to interpret as pollen lack morphologically diverse features to distinguish between closely related larch species [11].

Questions related to drivers of spatio-temporal changes of larch distributions need reliable discriminators between different Siberian larch species. The analysis of interspecific genetic variation has already proven successful in delimitating cryptic species [12,13], and could provide the means to identify larches to species or even population level. Moreover, a genetic approach could potentially be applied on ancient DNA from sediments [10,14], sub-fossil macro-remains [15,16], and even single pollen grains [17].

Ancient DNA is highly fragmented to sizes of less than 500 bp and accumulate chemical damage through time [18,19]. The probability that the target sequence is preserved over time is generally higher for organelle genomes because of their higher copy numbers per cell in comparison to the nuclear genome [14,20]. This makes organelle genomes preferential targets for ancient DNA studies. A genetic tool to identify larches to species level with simultaneous use for paleogenetic approaches in sediments has therefore several requirements: (1) it should be located on the chloroplast or the mitochondrial genome, (2) the diagnostic feature should focus on single nucleotide polymorphisms (SNPs), which are short and not too sensitive to sequencing errors, and (3) the target region must allow for the design of highly specific primer pairs that generate short amplicons and which avoid co-amplification of non-target genes and non-target species. Ideally, our target amplicon size should vary between 60 to 150 bp so that primers can also be used for High Resolution Melt Curve analysis to screen modern populations.

In *Larix*, like in most conifers of the Pinaceae family, the organelle genomes are haploid and inherited uniparentally. While the chloroplast genome is inherited paternally through pollen [21], the mitochondrial genome is inherited maternally [22] through the seeds. Therefore, the organelle genomes allow the distinction between paternal and maternal genealogies [23]. Structurally, the chloroplast genomes of larches are organized circularly and vary in size: 122,474 bp (*Larix decidua* Mill. [24], Accession: NC_016058.1), 122,492 bp (*Larix potaninii* var. *chinensis* (Voss) L.K.Fu & Nan Li [25], Accession: KX880508.1), 122,560 bp (*Larix sibirica* Ledeb., Accession: NC_036811.1), 122,583 bp (*Larix occidentalis* Nutt., Accession: MH612855.1 [26]) and 122,553–122,598 bp (*Larix gmelinii* var. *japonica* (Maxim. ex. Regel) Voss [27], Accession: LC228570 - LC228572). Mitochondrial genomes have a more complex organization than chloroplast genomes and contain between three to about 50 times their size in gymnosperms [28–31], but in spite of their size they carry only a few protein coding genes which are mainly components of the oxidative phosphorylation chain [32]. The largest part of the mitochondrial genome is non-coding and several studies have shown that a circular master-chromosome can coexist with (multiple) sub-genomic chromosomes as well as with linear plasmids [31,32]. Hence, only a few plant mitochondrial genomes are publicly available (search date 03.12.2018) with *Cycas taitungensis* C.F. Shen & al. [30], *Ginkgo biloba* L. [31], *Welwitschia mirabilis* Hook.f. [31], and 36 scaffolds of *Picea glauca* (Moench) Voss. [28] as the only gymnosperm representatives.

The aim of this study is to retrieve reference sequences for the detection of genetic variation between individuals from the tundra-taiga ecotone, covering longitudinally the ranges of *L*. *cajanderi* and *L*. *gmelinii* in northern Siberia. In particular, we aim to identify candidate SNPs suitable for the spatial discrimination of *Larix* in modern individuals as well as in ancient environmental samples. We sequenced and assembled the chloroplast genomes of 19 individuals from the northern distribution ranges of *L*. *gmelinii* (12 individuals) and *L*. *cajanderi* (7 individuals) in Siberia. As there was sufficient sequencing depth we additionally screened *de novo* assembled mitochondrial contiguous sequences (contigs) for SNPs. Our study presents new organelle genome reference sequences that allow the design of novel markers that can be the starting point for testing hypotheses, for example regarding *Larix* evolution, past and modern biogeography, and adaptive responses to changing environments.

## Material and methods

### Plant material

**Fig 1:**
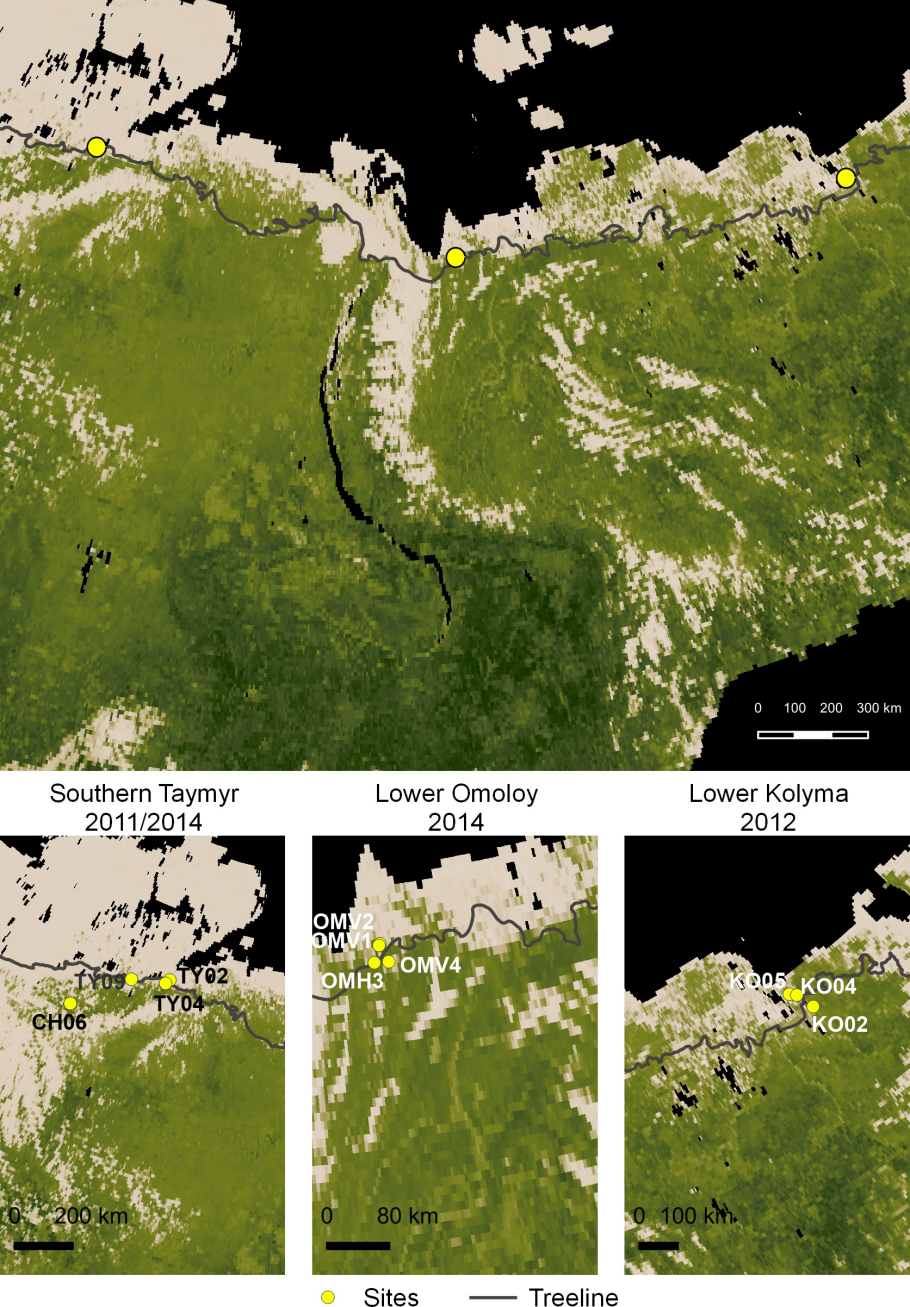
Map showing the sampled sites in northeastern Siberia. The Global Vegetation Map showing greenness in July 2014 was retrieved from NASA Earth Observations (https://earthobservatory.nasa.gov/global-maps/MOD_NDVI_M) and the treeline shape file was retrieved from the Circumpolar Arctic Vegetation Map [35].

Plant material was collected from forest-tundra transects in three regions located along a west-east gradient in North Siberia: on the southern Taymyr Peninsula (~97–105°E), Lower Omoloy River (~132°E), and Lower Kolyma River (~161°E) (Fig 1, Table 1). Sites were selected using satellite images and vegetation maps [33] to include uniform vegetation stands within three vegetation types: ‘single-tree tundra’, ‘forest-tundra’ (open stands at the northern forest margin), and ‘closed forest’ [34].

**Table 1:** List of the sequenced individuals with their NCBI accession number.

Sample code	Region	Site	Latitude (°N)	Longitude (°E)	Vegetation zone	Species range	Sample accession
EH103	Taymyr	TY02	72.54861	105.74611	single-tree stands*	*L*. *gmelinii*	MK468646
EH90	Taymyr	TY02	72.54896	105.74469	single-tree stands*	*L*. *gmelinii*	MK468638
EH91	Taymyr	TY02	72.54865	105.74546	single-tree stands*	*L*. *gmelinii*	MK468639
EH80	Taymyr	TY04	72.40889	105.44795	forest line	*L*. *gmelinii*	MK468633
EH83	Taymyr	TY04	72.40880	105.44791	forest line	*L*. *gmelinii*	MK468634
EH105	Taymyr	TY04	72.40881	105.44818	forest line	*L*. *gmelinii*	MK468648
EH84	Taymyr	TY09	72.14373	102.0624	forest line	*L*. *gmelinii*	MK468635
EH85	Taymyr	TY09	72.14367	102.06234	forest line	*L*. *gmelinii*	MK468636
EH86	Taymyr	TY09	72.14372	102.06265	forest line	*L*. *gmelinii*	MK468637
EH77	Taymyr	CH06	70.66506	97.70614	dense forest	*L*. *gmelinii*	MK468630
EH78	Taymyr	CH06	70.66496	97.70624	dense forest	*L*. *gmelinii*	MK468631
EH79	Taymyr	CH06	70.66485	97.70609	dense forest	*L*. *gmelinii*	MK468632
EH104	Omoloy	OMV1	70.74418	132.69852	forest line	*L*. *cajanderi*	MK468647
EH96	Omoloy	OMV2	70.72644	132.65817	forest line	*L*. *cajanderi*	MK468643
EH97	Omoloy	OMV4	70.52671	132.91426	dense forest	*L*. *cajanderi*	MK468644
EH98	Omoloy	OMH3	70.34396	132.90079	dense forest	*L*. *cajanderi*	MK468645
EH94	Kolyma	KO05	69.11839	161.02343	single trees on polygonal ridges	*L*. *cajanderi*	MK468642
EH92	Kolyma	KO04	69.05129	161.2066	open forest	*L*. *cajanderi*	MK468640
EH93	Kolyma	KO02	68.38994	161.449	dense forest	*L*. *cajanderi*	MK468641

Given are the sample codes of the individuals, their corresponding region of origin, the site codes, coordinates, vegetation zone, the published species range from which the individuals were collected. Krummholz individuals are marked with *.

In total, 19 trees were selected for investigation, which were vital, reproductive (indicated by cones), and at least 3.5 m tall, except for two krummholz individuals from the single-tree tundra (Table 1). Short twigs with needles were collected and placed in individual filter bags and dried on silica gel during fieldwork. For each of the four Omoloy sites and the three Lower Kolyma sites one individual per site was selected, while three individuals per site were sampled for the southern Taymyr Peninsula to allow both within and between site comparisons. A reference collection is available at the Alfred-Wegener-Institute Helmholtz Centre for Polar and Marine Research, Potsdam.

### DNA isolation and sequencing

We transferred 20 mg of needles per individual into impact-resistant 2 ml tubes together with two DNA-free steel beads of 5 mm diameter. The samples were cooled in liquid nitrogen for 3 minutes and ground to powder using FastPrep-24 (MP Biomedicals, USA) for 50 seconds at 4 m s^-1^. Total genomic DNA was isolated with the DNeasy Plant Mini Kit (Qiagen, Germany) according to the manufacturer’s protocol with two modifications: (1) we added 9 μl 1 M Dithiotreitol (VWR, Germany) to each sample during lysis after the RNase treatment and (2) elution was carried out twice with 100 μl molecular biology grade water (Omnilab, Germany), each with an incubation time of 5 minutes. The DNA quality was checked by gel electrophoresis and the DNA concentration was quantified with the Qubit dsDNA BR Assay on the Qubit 2.0 fluorometer (Invitrogen, USA).

The DNA samples were sent to StarSEQ sequencing service (Mainz, Germany) who performed the DNA library preparation, shotgun sequencing, and demultiplexing. Libraries were built with the TruSeq Nano DNA Library Prep Kit (Illumina, USA), during which each sample was given a distinct index to sequence several individuals in parallel (medium size of library = 500 bp). Paired-end sequencing (2 x 150 bp) was performed on an Illumina NextSeq 500 platform (Illumina, USA).

### Sequence processing and *de novo* assembly of chloroplast genomes

The quality check was conducted with FastQC [36] followed by trimming for quality and residual Illumina adapter sequences with Trimmomatic v. 0.3.2 [37] (settings: sliding window, window size = 4, average quality = 15, minimum quality to keep a base = 3, minimum length to keep a sequence = 40 nt). *De novo* assembly of each individual was carried out with CLC Genomics Workbench 8.0 (https://www.qiagenbioinformatics.com/) with an automatic word size, a bubble size of 50, and a minimum contig length of 200 nt. Afterwards, reads were mapped back to the contigs and the paired-end information was used to join contigs and build scaffolds. The scaffolds were aligned using the default settings with Geneious v 7.1.9 (http://www.geneious.com, [38]) to the *L*. *decidua* reference genome (Accession no.: NC_016058.1), the only complete published *Larix* reference at the time of the assembly (14.04.2015), to keep only those of putative chloroplast origin. The longest scaffolds (> 40,000 nt) of all individuals that aligned to the reference genome were overlapping without gaps in the alignment. The multiple alignment consensus resulted in a circular genome structure. Uncertain regions were re-sequenced (both inverted repeats including their adjacent regions, the transitions between contigs, and parts of *ycf*1). Therefore, we designed specific primer pairs using Primer3web v. 4.0.0 [39,40], with which we generated PCR products for Sanger sequencing (S1 Table). Furthermore, we designed a set of 18 primer pairs (S2 Table) and performed long-range PCRs followed by partial re-sequencing of the generated PCR products to validate the chloroplast genome structure (S3 Table, S1 Fig). The draft genome was then used for reference-guided assembly of the trimmed reads for each sequenced individual separately using the Burrows-Wheeler Aligner v. 0.7.12 (BWA-MEM default settings) [41], allowing the estimation of the coverage at each position of the genome.

### Chloroplast genome annotation and variant detection

The draft genome was annotated using cpGAVAS [42] and Geneious. Geneious implements a BLAST-like algorithm that transfers the annotation from the *L*. *decidua* reference genome based on a minimum similarity threshold of 70%. Transfer RNAs were annotated using tRNAscan-SE v. 1.21 [43,44]. The circular gene map was created with GenomeVx [45]. Correct positioning of start and stop codons of genes were checked by translating the coding sequences with codon translation table 11. A whole genome alignment of all 19 chloroplast genomes was computed using the progressiveMAUVE v. 2.3.1 [46,47] plugin in Geneious. Single nucleotide polymorphisms (SNPs) and insertions or deletions (InDels) of the individual genomes in comparison to the draft genome were checked through visual inspection, including the coverage at this position, and false base calls were manually corrected.

### Assembly of mitochondrial genomic sequences

For the identification of mitochondrial genomic sequences the complete mitochondrial reference genomes of land plants (Taxonomy ID: 3193) were downloaded from the National Center for Biotechnology Information Reference Sequence database (NCBI RefSeq, access date: 19.10.2017) [48] and combined with the 36 scaffolds of the *Picea glauca* mitochondrial genome [28]. The reference sequences were used to pre-filter the trimmed paired and unpaired reads for putative mitochondrial sequences using Bowtie2 v. 2.2.5 [49] with the preset-option “very sensitive” in the local alignment mode. SPAdes v. 3.6.2 [50] was used to test different k-mer sizes and choose the best for each individual for *de novo* assembly. BayesHammer [51], which is implemented in SPAdes was used for error correction and assembly statistics were produced with QUAST v. 3.1 [52]. The generated contigs (accessible under NCBI BioProject PRJNA528429) were then aligned against the 36 scaffolds of *Picea glauca* (NCBI accession numbers.: LKAM01000001.1- LKAM01000036.1), the closest published relative to the genus *Larix*, as well as to the *Cycas taitungensis* (NCBI accession No.: AP009381.1) mitochondrial genome using Geneious (mapping parameters: maximum gaps per read = 10%, maximum gap size = 50, word length = 24, maximum mismatches = 20%) and surveyed for SNPs. As our aim is the detection of SNPs that can be used in the screening of environmental and ancient samples, we avoided conceivably paralogous sequences as this would likely lead to unspecific amplification during PCR. In addition, the sequence alignment containing an SNP had to be unambiguously represented by at least 6 individuals and the second SNP variant had to be present at least three times to be robust or, in cases where the variant information was present for all individuals, at least two times.

### Analyses of genetic variation

In comparison to phylogenetic trees, haplotype networks allow for the introduction of loops and can thus display alternative genealogical relationships at the intraspecific population level with low divergence [53,54]. We estimated haplotype networks for the chloroplast (cpDNA) and mitochondrial (mtDNA) datasets respectively by computing absolute pairwise distances and used a statistical parsimony approach with TCS [53,54] implemented in PopART v. 1.7 (Population Analysis with Reticulate Trees [55]). Only SNPs were included in the distance matrix used for generating the haplotype networks.

## Results

### Chloroplast genome structure and genetic variation

**Fig 2:**
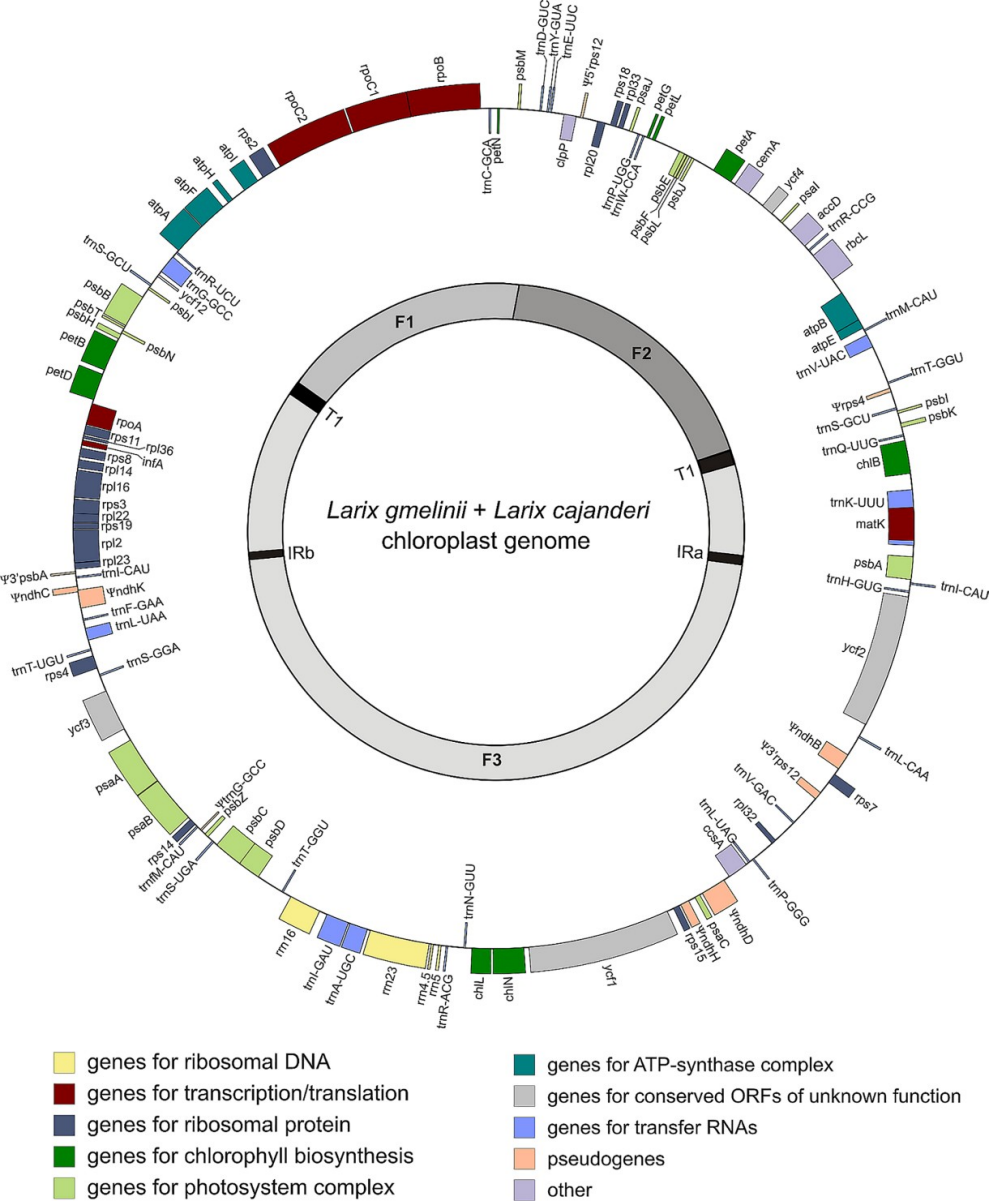
Circular gene map of the *L*. *gmelinii* and *L*. *cajanderi* chloroplast draft genome (122,590 nt). Genes outside the circle are transcribed clockwise while genes inside the circle are transcribed counter-clockwise. Pseudogenes are marked with Ψ.

Sequencing generated a total of 907,800,000 reads of which 80.8% passed trimming, and 62,338,218 paired reads showed the expected insert size and correct relative orientation. Maximum contig lengths obtained by the individual assemblies ranged between 43,219 nt and 117,053 nt. The draft chloroplast genome had a length of 122,590 nt resulting in a circular form with a GC-content of 38.74% (Fig 2). The sizes of the assembled chloroplast genomes differ slightly between 122,581 nt and 122,593 nt due to InDels and length differences of homopolymer stretches. Minimum coverage per base among all individual genomes varies between 35x (EH93-KO02) and 188x (EH80-TY04). The small single copy (SSC) region is composed of 56,387 nt and thus almost as long as the large single copy (LSC) region of 57,664 nt. Following Wu et al. [24] the *Larix* chloroplast can be structured into three regions of which the pseudogene Ψ*trnG*-GCC is defined as the border of the F2 region. This pseudogene was not predicted by tRNAscan-SE in the expected region, but between *psbZ* and *trnfM*-CAU. The retained but highly reduced inverted repeat (IR) regions are 306 nt long, containing only 3’*psbA* and *trnI*-CAU. A further inverted repeat of 481 nt length contains *psbI* and *trnS-*GCU and is located within the LSC. The genome encodes for 71 genes, 31 different transfer RNAs of which three are present with two copies, four ribosomal RNA genes, five putative genes of unknown function (*ycf*), and ten pseudogenes, which are mostly composed of NADH dehydrogenase pseudogenes (Table 2).

**Table 2:** Genes encoded by the *L*. *gmelinii* and *L*. *cajanderi* chloroplast genomes.

Group	Name of gene					
ribosomal RNA genes	*rrn4*.*5*	*rrn5*	*rrn16*	*rrn23*		
transfer RNA genes	*trnA-*UGC^a^	*trnC-*GCA	*trnD-*GUC	*trnE-*UUC	*trnF-*GAA	*trnfM-*CAU
	*trnG-*GCC	*trnH-*GUG	*trnI-*GAU^a^	*trnI-*CAU^b^	*trnK-*UUU^a^	*trnL-*CAA
	*trnL-*UAA^a^	*trnL-*UAG	*trnM-*CAU	*trnN-*GUU	*trnP-*GGG	*trnP-*UGG
	*trnQ-*UUG	*trnR-*ACG	*trnR-*CCG	*trnR-*UCU	*trnS-*GCU^b^	*trnS-*GGA
	*trnS-*TGA	*trnT-*GGT^b^	*trnT-*UGU	*trnV-*GAC	*trnV-*UAC^a^	*trnW-*CCA
	*trnY-*GTA					
30S ribosomal protein	*rps2*	*rps3*	*rps4*	*rps7*	*rps8*	*rps11*
	*rps14*	*rps15*	*rps18*	*rps19*		
50S ribosomal protein	*rpl2*^*a*^	*rpl14*	*rpl16*^*a*^	*rpl20*	*rpl22*	*rpl23*
	*rpl32*	*rpl33*	*rpl36*			
photosynthesis	*atpA*	*atpB*	*atpE*	*atpF*^*a*^	*atpH*	*atpI*
	*petA*	*petB*	*petD*^*a*^	*petG*	*petL*	*petN*
	*psaA*	*psaB*	*psaC*	*psaI*	*psaJ*	
	*psbA*	*psbB*	*psbC*	*psbD*	*psbE*	*psbF*
	*psbH*	*psbI*^*b*^	*psbJ*	*psbK*	*psbL*	*psbM*
	*psbN*	*psbT*	*psbZ*	*rbcL*		
chlorophyll biosynthesis	*chlB*	*chlL*	*chlN*			
transcription/translation component genes	*rpoA*	*rpoB*	*rpoC1*^*a*^	*rpoC2*	*infA*	*matK*
others	*accD*	*ccsA*	*cemA*	*clpP*		
unknown function	*ycf1*	*ycf2*	*ycf3*^*a*^	*ycf4*	*ycf12*	
pseudogenes	Ψ*ndhB*^*c*^	Ψ*ndhC*^*c*^	Ψ*ndhD*^*c*^	Ψ*ndhH*^*c*^	Ψ*ndhK*^*c*^	Ψ*rps4*^*c*^
	Ψ*rps12* (5’ end)^c^		Ψ*rps12* (3’ end)^c^		Ψ*psbA* (3’ end)^c^	
	Ψ*trnG*-GCC^c^					

^a^Genes with introns

^b^Genes with two copies

^c^Pseudogenes

Protein coding sequences account for 49.9% of the genome, transfer RNAs (tRNAs) for 2.1%, and ribosomal DNAs for 3.7%, while 44.3% of the genome is non-coding (S3 Table). Five genes (*rpoC1*, *petD*, *rpl16*, *rpl2*, *atpF*) and six tRNAs (*trnA*-UGC, *trnG*-GCC, *trnI*-GAU, *trnK*-UUU, *trnL-*UAA, *trnV*-UAC) contain one intron and *ycf3* contains two. The 31 tRNAs cover all amino acids. The tRNAs *trnI*-CAU and *trnT-*GGU have inverted repeats of each other while *trnG*-GCC has a pseudogene copy.

**Fig 3:**
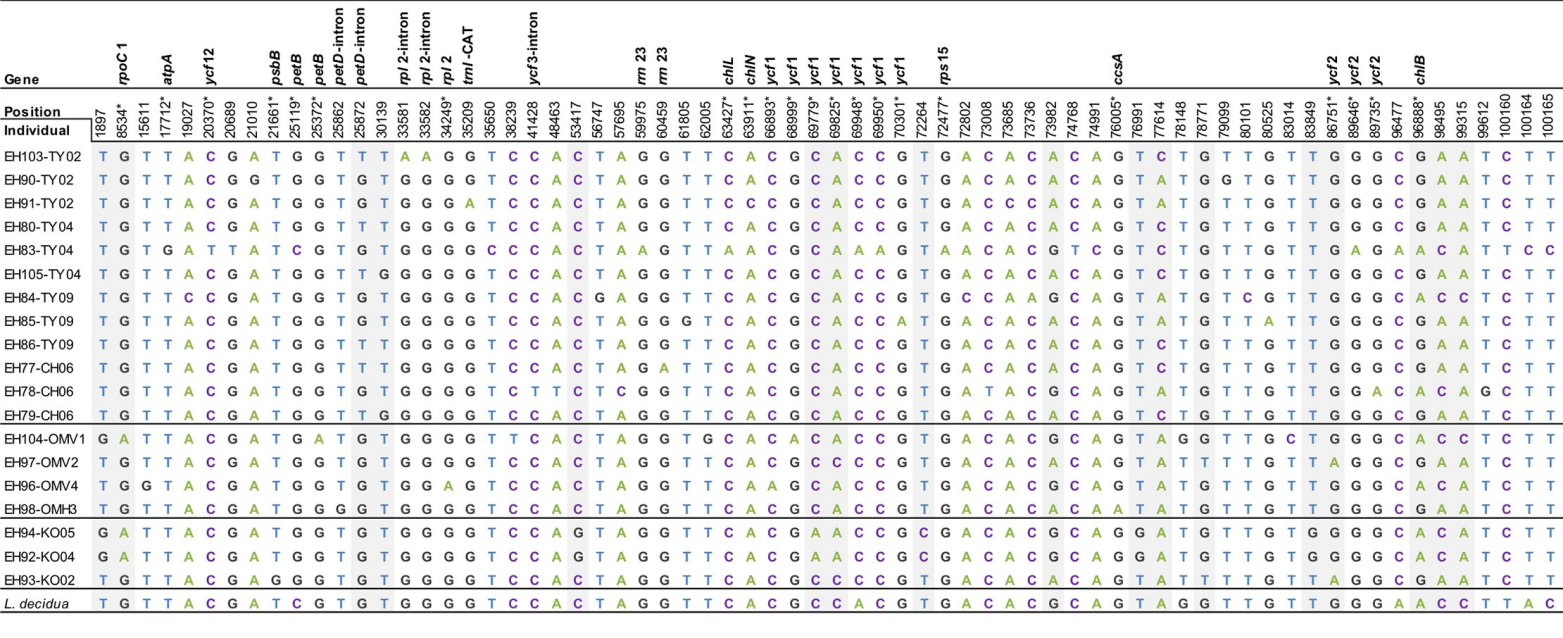
Concatenated alignment showing the 84 SNPs, with their position in the whole genome alignment of all sequenced individuals with the *L*. *decidua* reference. The 22 non-unique SNPs are shaded in gray. If a SNP is located within a gene or intron the corresponding gene name is given in the first row. Non-synonymous SNPs are marked by an asterisk (*). The nucleotides are shown in different colors (A = green, T = blue, C = purple, G = black).

The whole chloroplast genome alignment revealed 84 SNPs (Fig 3), one stem-loop inversion of 3 nt length (AGA↔TCT), five InDels (S 7), and 17 homopolymer stretch differences (12x poly(T), 4x poly(A), 1x poly(C)). More than half of the SNPs (56%) are transversions (25% A↔C, 2.4% A↔T, 26.2% G↔T, 2.4% G↔C) while 44% of the SNPs are transitions (25% A↔G, 19% C↔T). The majority of the SNPs occurred only once (singletons). There are 27 SNPs located in coding regions, one of these in a transfer RNA gene. Out of the SNPs in coding regions, 8 result in a change of the translated amino acid sequence of which, in three cases, the amino acid property is also changed (Table 3). The two genes *ycf1* and *ycf2* encompass 10.5% of the whole genome, but contain 11.9% of all SNPs. In non-coding regions we detected 57 SNPs, of which 41 were singletons. The number of pairwise differences between the chloroplast genomes ranges from a minimum of 0 between EH92-KO04 and EH94-KO05 to a maximum of 57 between EH83-TY04 and EH84-TY09. Individuals from the southern Taymyr Peninsula exhibit less (between 1 and 57, mean 19) pairwise differences in comparison with those from the Omoloy and Kolyma regions (between 0 and 34, mean 23). Among all individuals, EH83-TY04 has the most pairwise differences to all other individuals due to 21 unique SNPs (Fig 3).

**Table 3:** Chloroplast genes with non-synonymous SNPs, their position in the protein sequence and changes in amino acid properties.

Gene	Position	Change	Amino acid property changes
*accD*	91	Arginine (R)—Cysteine (C)	Basic—Cytosine
*atpA*	139	Serine (S)—Alanine (A)	Hydrophile—hydrophile
*chlL*	282	Glutamine (Q)—Lysine (K)	Acid/amid—basic
*petL*	44	Lysine (K)—Glutamine (Q)	Basic—Acid/amid
*rps15*	63	Valine (V)—Isoleucine (I)	Hydrophobe—hydrophobe
*ycf1*	1525	Isoleucine (I)—Leucine (L)	Hydrophobe—hydrophobe
*ycf1*	1559	Leucine (L)—Isoleucine (I)	Hydrophobe—hydrophobe
*ycf2*	459	Proline (P)—Serine (S)	Hydrophile—hydrophile

**Fig 4:**
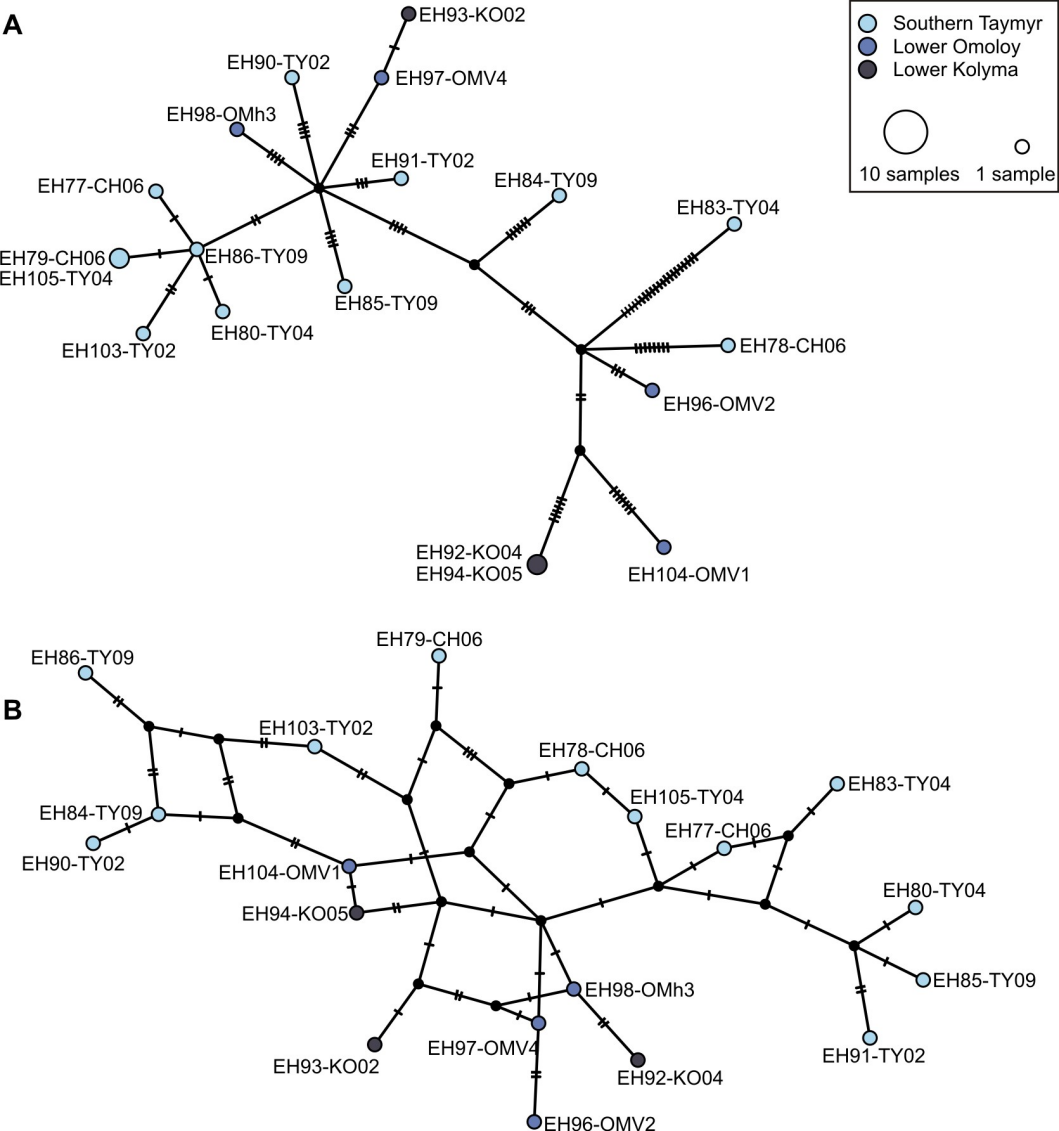
**Statistical parsimony haplotype networks for (A) chloroplast single nucleotide polymorphisms (SNPs) and (B) mitochondrial SNPs with haplotypes as circles, whose size is proportional to the number of individuals exhibiting this haplotype.** Circles are colored according to their region of origin while small black circles represent estimated putative intermediate haplotypes. Hatch marks along the edges indicate mutations between the nodes. Loops indicate alternative pathways in the genealogy.

The cpDNA based TCS haplotype network shows no spatial structure between haplotypes collected from the Taymyr, Omoloy, and Kolyma regions (Fig 4A). The haplotypes are divided into two groups with one intermediate haplotype (EH84-TY09) between both groups. Haplotypes of the first group, shown in the upper part of the haplotype network, exhibit 43 unique SNPs, which correspond to 51.2% of all detected SNPs. The haplotypes of the second group, shown in the lower part of the haplotype network, are more closely related to each other in comparison to the haplotypes of the first group and display 18 singletons (21% of all SNPs).

A whole chloroplast genome alignment, which included not only the individuals sequenced in this study, but also all available complete *Larix* chloroplast genomes on NCBI, was then used to compare the SNP variants detected in this study with different *Larix* species. In the majority of non-singleton SNPs the haplotypes of the group shown in the upper part of the network (Fig 4A) carry the same variant as other *Larix* species whereas the group in the lower part of the network carries the potentially derived variant. At two positions—one in the 4.5S rRNA gene and one at a non-coding site—can the individuals sequenced in this study be distinguished from all other *Larix* genomes in the alignment, except for EH83-TY04, which shares the putatively ancestral variant at these positions.

### Genetic variation in mitochondrial sequences

For each individual we retrieved between 1,728 and 5,000 assembled contigs of maximum sizes between 5,356 and 18,252 bp. A total of 52,765 contigs were generated of which 15,576 aligned to the *Picea glauca* scaffolds, except for scaffolds 28, 31, 35, and 36 where none of the assembled contigs aligned. A general problem was that only relatively short contigs aligned, leaving large gap stretches. The mapping resulted in the retrieval of 59 mostly partial genes (Table 4).

**Table 4:** Genes detected among *L*. *gmelinii* and *L*. *cajanderi* mitochondrial sequences.

Group	Name of genes				
**Respiratory chain**					
Complex I (NADH dehydrogenase)	*nad1*	*nad2*	*nad3*	*nad4*	*nad4a*
	*nad4L*	*nad5*	*nad6*	*nad7*	*nad9*
Complex II (Succinat dehydrogenase)	*sdh3*				
Complex III (Cytochrome c reductase)	*cob*				
Complex IV (Cytochrome c oxidase)	*cox1*	*cox2*	*cox3*		
Complex V (ATP synthase)	*atp1*	*atp4*	*atp6*	*atp8*	*atp9*
Cytochrome c maturation factors	*ccmB*	*ccmC*	*ccmFC1*	*ccmFC2*	*ccmFN*
**Ribosomal protein**					
large subunit	*rpl10*	*rpl16*	*rpl2*	*rpl5*	
small subunit	*rps1*	*rps10*	*rps11*	*rps12*	*rps13*
	*rps14*	*rps19*	*rps2*	*rps3*	*rps4*
	*rps7*				
**rRNA**	*rrn18*	*rrn26*	*rrn5*		
**tRNA**	*trnC*-GCA	*trnD-GUC*	*trnE-UUC*	*trnH-GUG*	*trnM-CAU*
	*trnN*-GUU	*trnP-AGG*	*trnP-UGG*	*trnQ-UUG*	*trnR-UCG*
	*trnW-CCA*	*trnY-GUA*			
**Photosystem I**	*psaA*				
**others**	*matR*	*mttB*			
**putative gene with unidentified function**	*ymfcob*				

Among the mapped contigs, we detected a total of 213 SNPs, of which 172 were detected in non-coding regions (S4 Table). At 8 non-coding positions, we detected SNPs with one variant present only in individuals from the southern Taymyr Peninsula and the other variant only in those from the Lower Omoloy and Lower Kolyma regions (Table 5). Unfortunately these SNP positions are covered only by some of the individuals. We detected 16 SNPs which were represented by all sequenced individuals and were used to compute a statistical parsimony-based haplotype network (Fig 4B). Haplotypes within the Omoloy population are mostly closely related to each other as indicated by their close proximity in the haplotype network. The haplotypes of the Kolyma populations are more closely related to the Omoloy haplotypes than to each other, as the Omoloy haplotypes are placed between them. From the Taymyr population most haplotypes are relatively closely related and connected with each other displaying alternative genealogical pathways. According to the network, the Taymyr haplotypes form two distinctive groups, with the Omoloy and Kolyma haplotypes between them.

**Table 5:** List of putatively diagnostic mitochondrial SNPs.

scaffold	1	4	6	6	11	15	17	20
position	1023485	65316	194010	325675	95116	5654	36760	27703
**EH77-CH06**	A	G	-	C	A	A	-	A
**EH78-CH06**	A	G	A	C	A	A	C	A
**EH79-CH06**	A	-	A	C	-	A	-	-
**EH80-TY04**	A	G	A	C	A	A	-	A
**EH83-TY04**	A	G	A	-	A	A	C	-
**EH105-TY04**	-	G	A	-	A	A	-	-
**EH84-TY09**	A	-	-	-	A	-	C	-
**EH85-TY09**	A	G	A	-	A	A	-	-
**EH86-TY09**	A	-	-	C	A	A	C	-
**EH90-TY02**	A	-	A	C	-	A	C	A
**EH91-TY02**	A	G	A	C	A	A	-	A
**EH103-TY02**	A	-	A	C	-	A	-	A
**EH92-KO04**	C	T	C	A	G	-	T	C
**EH93-KO02**	C	T	C	-	G	-	-	-
**EH94-KO05**	C	T	C	A	G	C	T	C
**EH96-OM2**	C	T	C	A	G	C	-	-
**EH97-OM4**	C	T	C	A	G	C	T	C
**EH98-OMh3**	C	-	C	A	G	C	T	C
**EH104-OM1**	C	T	-	A	G	C	T	C
***Picea glauca***	G	G	C	C	A	A	C	A

The location of the SNPs is given by the scaffold of the *Picea glauca* reference genome and the position on the scaffold in the alignment. For each individual as well as the reference genome the corresponding SNP variant is shown if sequence information was present.

## Discussion

### *De novo* assembly of chloroplast genomes and mitochondrial sequences

In this study 55.7% of the *Larix* chloroplast genome was composed of coding sequences, which is comparable to other conifers (e.g. *L*. *decidua*: 56.0% coding [24], *Picea* spp. on average coding: 55.2% [56]). The structural arrangement and gene content of the assembled chloroplast genomes are both similar to *L*. *decidua*, which facilitated the contig arrangement in the assembly process and annotation, and is in agreement with Wu et al. [24] who showed that the genus *Larix* has a distinct structure within the Pinaceae family. The overall size of the generated draft genome (122,590 nt) is 116 nt larger than the sequence of *L*. *decidua*, while the IR regions (306 nt), which are typically reduced in Pinaceae, are smaller than those of *L*. *decidua* (436 nt) [24], *L*. *potaninii* var. *chinensis* (435 nt) [25], *Picea morrisonicola* (440 nt), and *Pinus wilsoniana* (345 nt), but larger than those of *Cedrus deodara* (236 nt) and *Keteleeria davidiana* (267 nt) [57].

The mitochondrial genome could not be fully recovered despite its multi-copy presence within plant cells. With regard to gymnosperms, the mitochondrial genomes of *Picea glauca* (5.9 Mb [28]) and *Picea abies* (> 4.3 Mb [29]) are amongst the largest ones that have been published for land plants ([29]), followed in size by *Welwitschia mirabilis* (979 kb [31]), whereas *Cycas taitungensis* (414 kb [30]) and *Ginkgo biloba* (347 kb [31]) are much smaller. Still, their sizes suggest that the mitochondrial genome of *Larix* is also quite large and might contain a high structural complexity. The generated contigs aligned only in a few conserved parts scattered across the scaffolds of *P*. *glauca* and even less across the *C*. *taitungensis* reference genome, suggesting that most parts of the *Larix* mitochondrial genome have evolved differently. The contigs were short overall and those which aligned to the *P*. *glauca* genome mostly had a mean coverage below 5x. If the complete mitochondrial genome is the aim of the sequencing, the sample could be enriched for mitochondria and/or a larger sequencing depth would be necessary. Furthermore, paired-end sequencing could be coupled with mate-pair sequencing following, for example, Jackman et al. [28]. Many sequences orthologous to *P*. *glauca* displayed two different versions of the sequences (with small InDels and several SNPs) and both sequence variants were often represented by the same individual, indicating false alignment of paralogous sequences. These were discarded as such parts would inflate nucleotide diversity and result in a wrong phylogeny [58,59].

In comparison to the genetic variation in coding regions of the chloroplast, less genetic variation was found in mitochondrial coding regions. A highly efficient repair mechanism is currently proposed as the reason for the generally low mutation rate in plant mitochondrial genomes [11]. Nevertheless, we retrieved 172 SNPs in non-coding regions of putative mitochondrial origin, making the small amount of new sequence information highly valuable. For example, it can be used to design primers or hybridization probes to screen the identified polymorphisms not only on a broader geographic scale in extant populations, but also back in time using permafrost or lake sediment cores, for example, as recently shown by Epp et al. [10] and Zimmermann et al. [60].

### SNP detection of *Larix* at the tundra-taiga ecotone and marker evaluation for paleogenetic investigations

For *L*. *gmelinii* and *L*. *cajanderi* various molecular markers have been used to infer genetic structure among extant populations [7,23,61–63], identify glacial refugia [23], or to investigate the phylogenetic relationship of the genus [64–66]. *Larix* populations at the tundra-taiga ecotone though, remain under-represented or are not considered at all in these studies, probably due to the remoteness and difficult accessibility of these regions. Recent and past dynamics in these boundary regions are therefore largely unknown. Many of these studies had to rely on references of distantly-related taxa for their marker design, since species-specific reference genomes for these dominant Asian larch species are not available. Our chloroplast and mitochondrial genomic data can therefore provide a basis for the development of new genetic markers to analyze larch population dynamics at various spatio-temporal scales.

The detected number of SNPs comprised approximately 0.07% of the draft genome. This is to be expected in closely related species such as *Larix*, because the major part of the genome comprises coding sequence information for physiologically important processes of the photosynthesis machinery [67] and is thus essential for survival. Approximately 8% of all variable positions are located in the *ycf1* region, whose elevated substitution rate has been reported in several plant species before (among conifers:[24,25,57]) and even proposed as a putative plant plastid barcode [68]. The future applicability of this region as a genetic marker for *Larix* is, however, limited, since highly repetitive motifs, inversions, and large InDels prevent the design of specific primer pairs to produce short amplicons. Regardless, the comparison with publicly available whole chloroplast genomes revealed four positions where the 19 sequenced individuals display a different variant, only with the exception of individual EH83-13TY04. In the future these SNPs could be evaluated for their potential as diagnostic markers to distinguish individuals from the range of *L*. *gmelinii*/*L*. *cajanderi* from other *Larix* species in hybridization zones. Officially, *rbcL* and *matK* are the recommended plant barcoding genes [69,70] while the short P6-loop of the *trnL*-UAA intron [71] is commonly used for vascular plant DNA metabarcoding, for example from ancient and modern sediments [10,11,72,73] or animal diet [74,75]. Across *matK*, two SNPs were detected that might be diagnostic for either *L*. *sibirica* or *L*. *occidentalis*, whereas all other compared sequences were identical. The short P6-loop of the *trnL*-UAA intron shows only one known polymorphic site in *L*. *sibirica* that could be diagnostic for this species [76]. Only by using the complete *rbcL* gene sequence (size: 1428 bp) is it possible to distinguish the published genomes from the ones generated in this study, but it is not possible to spatially separate the individuals sequenced here from each other or even from *L*. *gmelinii* var. *olgensis*. This is, however, not surprising as even the complete chloroplast genomes from the sequenced individuals cannot be used to separate them geographically.

The mitochondrial haplotype network showed at least some spatial structure with haplotypes from the Omoloy and Kolyma populations being more closely related to each other than to most of the haplotypes from the Taymyr populations. Although haplotypes from the Taymyr populations seem to have diverged into two groups with haplotypes from the Omoloy and Kolyma regions lying between them, we detected 8 SNPs that displayed one variant in individuals sampled in the Taymyr region while the other variant occurred only in those from the Omoloy and Kolyma regions. As these SNPs are not supported by coverage from all individuals and as our sample size is very low their applicability as a potentially diagnostic marker needs to be tested. This would still not resolve the lack of spatial separation of *Larix* populations at the northern distribution limit.

The absence of a clear spatial structure in the chloroplast genome and, for the Taymyr individuals, the mitochondrial genome is in contrast to our expectations. First, *L*. *gmelinii* and *L*. *cajanderi* have been suggested to be a progenitor-derivative species pair [7,77]. Second, as mitochondrial genomes are transmitted through seeds, and parentage analysis of *Larix* nuclear microsatellites from the southern Taymyr Peninsula suggest median seed dispersal distances of about 10 m [78], we anticipated a clear spatial structure at least based on mitochondrial polymorphisms. Third, even though chloroplast DNA is wind-dispersed by pollen, it is astonishing that no spatial structure can be found over such a vast longitudinal distance, which ultimately leads to the question, how is this possible? The longevity of *Larix* trees, their predominantly outcrossing mating system, the wind-dispersed pollen, and their life-long reproductive capacity after reaching maturity are strategies to maintain a high genetic diversity within populations [79]. Furthermore, *Larix* has large population sizes in Siberia without any competition from evergreen conifers and broadleaved trees over most of its range. Typically, these traits result in a higher genetic diversity within populations than among them in comparison to other growth forms [79]. This was recently shown for *Larix* on the southern Taymyr Peninsula in a study based on nuclear microsatellite markers, where low genetic differentiation of sub-populations was indicative of region-wide high gene flow [80], despite their relatively low pollen productivity and proclaimed limited dispersal capacity in comparison to other Pinaceae species with wind-dispersed pollen [81,82]. Furthermore, Polezhaeva et al. [23] who also found shared chloroplast and mitochondrial haplotypes among *Larix* across northeast Asia based on chloroplast single sequence repeats (SSR) and mitochondrial restriction fragment length polymorphisms (RFLP), suggest that large population sizes and long generation times could promote the sharing of ancestral polymorphism among different *Larix* species. This might explain the lack of spatial structure for the chloroplast genomes.

The southern Taymyr Peninsula has been described as a possible hybridization zone between *L*. *gmelinii* and *L*. *sibirica*. Semerikov et al. [7] propose that *L*. *gmelinii* expanded into the range of *L*. *sibirica* after the Last Glacial Maximum. The neutral model of Currat et al. [83], predicts that introgression follows predominantly from the established species to the invading species and affects mostly organelle genomes experiencing low gene flow, such as the mitochondrial genome. Based on this assumption we hypothesize that the four individuals showing more distantly related haplotypes in the upper part of the mitochondrial haplotype network that also carry the *L*. *gmelinii*/*L*. *cajanderi* chloroplast genome might be either hybrids between *L*. *gmelinii* and *L*. *sibirica* or they might be introgressed after back-crossing with one of the parental species that hybridized. This could be tested in the future by analyzing nuclear genetic variation in these individuals [23,84], as the nuclear genome of *L*. *sibirica* has just been published [85] and work regarding the mitochondrial genome is in progress.

Finally, the chloroplast haplotypes display two star-like patterns in the lower part of the network. Star-like patterns are usually deduced as a result of population expansion following Slatkin and Hutchinson [86]. Consequently, the pattern could point towards two separate population expansion events, which is a further hypothesis that can be explored in future investigations.

### Conclusions and future directions

The Russian Far Northeast is characterized by notable environmental changes over a wide range of time-scales that probably originate from complex interactions between climate, permafrost and biota along with time-lags [87] and feedback mechanisms [88]. Genetic signatures of modern populations are only a snapshot in time and can thus only provide a limited understanding about past processes, the identification of glacial refugia, or the gain and loss of genetic variability. Natural archives such as permafrost or lake sediments often contain pollen or macrofossil evidence of *Larix* [8,9,89,90] and, even in the absence of macrofossils, traces of ancient DNA can be found. The *de novo* assembled organelle reference genomes, the novel polymorphisms we detected, and the derived chloroplast and mitochondrial haplotype networks presented in this study could be the starting point for more detailed investigations by screening modern populations across northeast Asia, and by screening ancient *Larix* DNA from sediments, single pollen grains, or macrofossils. Therefore, the generated sequence information provides a basis to design more specific genetic markers and probes for the targeted enrichment of the desired sequences from ancient samples. This would allow tracing haplotype changes not only in space but also back in time, which in turn would allow hypothesis testing, for example about the timing and extent of glacial range contractions and post-glacial population expansions including their genetic consequences (founder events, bottleneck effects) or about the biogeographic history, including possible competition between different *Larix* species during range expansions [7,10].

## Supporting information

S1 Fig Map showing the position of primer pairs for long-range PCRs. The outer circle shows the positions of the 18 primer pairs for long-range PCRs. The second circle shows the re-sequenced parts of the chloroplast genomes after long-range PCR. The innermost circle represents the length of the *Larix gmelinii* and *Larix cajanderi* chloroplast genome in kilo base pairs (kb).
(PDF)

S1 Table Primer sequences for validation of inverted repeat placement and uncertain regions. (XLSX)

S2 Table Primer sequences for long-range PCRs. (XLSX)

S3 Table Nucleotide distribution of the *Larix gmelinii/Larix cajanderi* draft chloroplast genome. (XLSX)

S4 Table List of mitochondrial SNPs. For each of the 213 SNP the NCBI accession number of the reference genome is given with its position in the mapping. For coding parts the orientation of the gene in the reference genome is given.
(XLSX)

## References

[1] Abaimov AP. Geographical distribution and genetics of Siberian larch species Permafrost Ecosystems: Siberian Larch Forests. Dordrecht; New York: Springer; 2010 pp. 41–58.

[2] Forest Fund of Russia. Book for Forest Fund of the Russian Federation Rosleskhos, Moscow: All-Russia Research and Information Center for Forest Researches, Federal Forest Service; 1999.

[3] Frost GV, Epstein HE. Tall shrub and tree expansion in Siberian tundra ecotones since the 1960s. Glob Change Biol. 2014;20: 1264–1277. 10.1111/gcb.12406 24115456

[4] Kruse S, Wieczorek M, Jeltsch F, Herzschuh U. Treeline dynamics in Siberia under changing climates as inferred from an individual-based model for *Larix*. Ecological Modelling. 2016;338: 101–121. 10.1016/j.ecolmodel.2016.08.003

[5] MacDonald G., Kremenetski K., Beilman D. Climate change and the northern Russian treeline zone. Philosophical Transactions of the Royal Society B: Biological Sciences. 2008;363: 2283–2299. 10.1098/rstb.2007.2200 18006415 PMC2606780

[6] Callaghan TV, Werkman BR, Crawford RMM. The tundra-taiga interface and its dynamics: concepts and applications. Ambio. 2002;Special Report 12: 6–14. 12374061

[7] Semerikov VL, Semerikova SA, Polezhaeva MA, Kosintsev PA, Lascoux M. Southern montane populations did not contribute to the recolonization of West Siberian Plain by Siberian larch (Larix sibirica): a range-wide analysis of cytoplasmic markers. Mol Ecol. 2013;22: 4958–4971. 10.1111/mec.12433 24033458

[8] Kienast F, Wetterich S, Kuzmina S, Schirrmeister L, Andreev AA, Tarasov P, et al Paleontological records indicate the occurrence of open woodlands in a dry inland climate at the present-day Arctic coast in western Beringia during the Last Interglacial. Quaternary Science Reviews. 2011;30: 2134–2159. 10.1016/j.quascirev.2010.11.024

[9] Klemm J, Herzschuh U, Pestryakova LA. Vegetation, climate and lake changes over the last 7000 years at the boreal treeline in north-central Siberia. Quaternary Science Reviews. 2016;147: 422–434. 10.1016/j.quascirev.2015.08.015

[10] Epp LS, Kruse S, Kath NJ, Stoof-Leichsenring KR, Tiedemann R, Pestryakova LA, et al Temporal and spatial patterns of mitochondrial haplotype and species distributions in Siberian larches inferred from ancient environmental DNA and modeling. Scientific Reports. 2018;8: 17436 10.1038/s41598-018-35550-w 30498238 PMC6265258

[11] Niemeyer B, Epp LS, Stoof-Leichsenring KR, Pestryakova LA, Herzschuh U. A comparison of sedimentary DNA and pollen from lake sediments in recording vegetation composition at the Siberian treeline. Mol Ecol Resour. 2017; 1–17. 10.1111/1755-0998.12636 28488798

[12] Stoof-Leichsenring KR, Epp LS, Trauth MH, Tiedemann R. Hidden diversity in diatoms of Kenyan Lake Naivasha: a genetic approach detects temporal variation. Molecular Ecology. 2012;21: 1918–1930. 10.1111/j.1365-294X.2011.05412.x 22221342

[13] Dainou K, Blanc-Jolivet C, Degen B, Kimani P, Ndiade-Bourobou D, Donkpegan ASL, et al Revealing hidden species diversity in closely related species using nuclear SNPs, SSRs and DNA sequences—a case study in the tree genus *Milicia*. BMC Evol Biol. 620;16: 259 10.1186/s12862-016-0831-9 27903256 PMC5131513

[14] Parducci L, Jørgensen T, Tollefsrud MM, Elverland E, Alm T, Fontana SL, et al Glacial survival of boreal trees in northern Scandinavia. Science. 2012;335: 1083–1086. 10.1126/science.1216043 22383845

[15] Lendvay B, Balint M, Pal I, Vincze I, Orban I, Magyari EK. Plant macrofossils from lake sediment as the material to assess ancient genetic diversity: Did deforestation influence Norway spruce (Picea abies) in the South Carpathians? Quat Int. 2018;477: 106–116. 10.1016/j.quaint.2018.02.023

[16] Wagner S, Litt T, Sanchez-Goni M-F, Petit RJ. History of Larix decidua Mill. (European larch) since 130 ka. Quat Sci Rev. 2015;124: 224–247. 10.1016/j.quascirev.2015.07.002

[17] Parducci L, Suyama Y, Lascoux M, Bennett KD. Ancient DNA from pollen: a genetic record of population history in Scots pine. Molecular Ecology. 2005;14 10.1111/j.1365-294X.2005.02644.x 16029485

[18] Hofreiter M, Serre D, Poinar HN, Kuch M, Pääbo S. Ancient DNA. Nature Reviews Genetics. 2001;2: 353–359. 10.1038/35072071 11331901

[19] Pääbo S. Ancient DNA: extraction, characterization, molecular cloning, and enzymatic amplification. Proc Natl Acad Sci USA. 1989;86: 1939–1943. 10.1073/pnas.86.6.1939 2928314 PMC286820

[20] Parducci L, Bennett KD. The real significance of ancient DNA. American Journal of Botany. 2017;104: 800–802. 10.3732/ajb.1700073 28588137

[21] Szmidt AE, Aldén T, Hällgren J-E. Paternal inheritance of chloroplast DNA in *Larix*. Plant Molecular Biology. 1987;9: 59–64. 10.1007/BF00017987 24276798

[22] DeVerno LL, Charest PJ, Bonen L. Inheritance of mitochondrial DNA in the conifer *Larix*. Theoretical and Applied Genetics. 1993;86: 383–388. 10.1007/BF00222106 24193487

[23] Polezhaeva MA, Lascoux M, Semerikov VL. Cytoplasmic DNA variation and biogeography of *Larix* Mill. in Northeast Asia. Molecular Ecology. 2010;19: 1239–1252. 10.1111/j.1365-294X.2010.04552.x 20163546

[24] Wu C-S, Lin C-P, Hsu C-Y, Wang R-J, Chaw S-M. Comparative chloroplast genomes of Pinaceae: insights into the mechanism of diversified genomic organizations. Genome Biol Evol. 2011;3: 309–319. 10.1093/gbe/evr026 21402866 PMC5654405

[25] Han K, Li J, Zeng S, Liu Z-L. Complete chloroplast genome sequence of Chinese larch (Larix potaninii var. chinensis), an endangered conifer endemic to China. Conservation Genet Resour. 2016; 1–3 10.1007/s12686-016-0633-9

[26] Gernandt DS, Arias CR, Terrazas T, Dugua XA, Willyard A. Incorporating fossils into the Pinaceae tree of life. American Journal of Botany. 2018;105: 1329–1344. 10.1002/ajb2.1139 30091785

[27] Ishizuka W, Tabata A, Ono K, Fukuda Y, Hara T. Draft chloroplast genome of *Larix gmelinii* var. *japonica*: insight into intraspecific divergence. Journal of Forest Research. 2017;0: 1–6. 10.1080/13416979.2017.1386019

[28] Jackman SD, Warren RL, Gibb EA, Vandervalk BP, Mohamadi H, Chu J, et al Organellar genomes of White Spruce (Picea glauca): assembly and annotation. Genome Biol Evol. 2015;8: 29–41. 10.1093/gbe/evv244 26645680 PMC4758241

[29] Nystedt B, Street NR, Wetterbom A, Zuccolo A, Lin Y-C, Scofield DG, et al The Norway spruce genome sequence and conifer genome evolution. Nature. 2013;497: 579–584. 10.1038/nature12211 23698360

[30] Chaw S-M, Shih AC-C, Wang D, Wu Y-W, Liu S-M, Chou T-Y. The mitochondrial genome of the gymnosperm Cycas taitungensis contains a novel family of short interspersed elements, Bpu sequences, and abundant RNA editing sites. Mol Biol Evol. 2008;25: 603–615. 10.1093/molbev/msn009 18192697

[31] Guo W, Grewe F, Fan W, Young GJ, Knoop V, Palmer JD, et al *Ginkgo* and *Welwitschia* mitogenomes reveal extreme contrasts in gymnosperm mitochondrial evolution. Molecular Biology and Evolution. 2016;33: 1448–1460. 10.1093/molbev/msw024 26831941

[32] Gualberto JM, Mileshina D, Wallet C, Niazi AK, Weber-Lotfi F, Dietrich A. The plant mitochondrial genome: dynamics and maintenance. Biochimie. 2014;100: 107–120. 10.1016/j.biochi.2013.09.016 24075874

[33] Schlesinger P, Stone TA. RLC vegetative cover of the former Soviet Union, 1990 Oak Ridge National Laboratory Distributed Active Archive Center, Oak Ridge, Tennessee, USA; 2004.

[34] Wieczorek M, Kruse S, Epp LS, Kolmogorov A, Nikolaev AN, Heinrich I, et al Dissimilar responses of larch stands in northern Siberia to increasing temperatures—a field and simulation based study. Ecology. 2017;98: 2343–2355. 10.1002/ecy.1887 28475233

[35] CAVM Team. Circumpolar Arctic Vegetation Map (1:7,500,000 scale), Conservation of Arctic Flora and Fauna (CAFF) Map No. 1. U.S. Fish and Wildlife Service, Anchorage, Alaska [Internet]. 2003. Available: http://www.geobotany.uaf.edu/cavm/

[36] Andrews S. FastQC: a quality control tool for high throughput sequence data [Internet]. 2010. Available: http://www.bioinformatics.babraham.ac.uk/projects/fastqc/

[37] Bolger AM, Lohse M, Usadel B. Trimmomatic: a flexible trimmer for Illumina sequence data. Bioinformatics. 2014;30: 2114–2120. 10.1093/bioinformatics/btu170 24695404 PMC4103590

[38] Kearse M, Moir R, Wilson A, Stones-Havas S, Cheung M, Sturrock S, et al Geneious Basic: An integrated and extendable desktop software platform for the organization and analysis of sequence data. Bioinformatics. 2012;28: 1647–1649. 10.1093/bioinformatics/bts199 22543367 PMC3371832

[39] Koressaar T, Remm M. Enhancements and modifications of primer design program Primer3. Bioinformatics. 2007;23: 1289–1291. 10.1093/bioinformatics/btm091 17379693

[40] Untergasser A, Cutcutache I, Koressaar T, Ye J, Faircloth BC, Remm M, et al Primer3—new capabilities and interfaces. Nucleic Acids Research. 2012;40: e115 10.1093/nar/gks596 22730293 PMC3424584

[41] Li H, Durbin R. Fast and accurate short read alignment with Burrows–Wheeler transform. Bioinformatics. 2009;25: 1754–1760. 10.1093/bioinformatics/btp324 19451168 PMC2705234

[42] Liu C, Shi L, Zhu Y, Chen H, Zhang J, Lin X, et al CpGAVAS, an integrated web server for the annotation, visualization, analysis, and GenBank submission of completely sequenced chloroplast genome sequences. BMC Genomics. 2012;13: 715 10.1186/1471-2164-13-715 23256920 PMC3543216

[43] Lowe TM, Eddy SR. tRNAscan-SE: A Program for improved detection of transfer RNA genes in genomic sequence. Nucl Acids Res. 1997;25: 0955–0964. 10.1093/nar/25.5.0955 PMC146525 9023104

[44] Schattner P, Brooks AN, Lowe TM. The tRNAscan-SE, snoscan and snoGPS web servers for the detection of tRNAs and snoRNAs. Nucl Acids Res. 2005;33: W686–W689. 10.1093/nar/gki366 15980563 PMC1160127

[45] Conant GC, Wolfe KH. GenomeVx: simple web-based creation of editable circular chromosome maps. Bioinformatics. 2008;24: 861–862. 10.1093/bioinformatics/btm598 18227121

[46] Darling AE, Mau B, Perna NT. progressiveMauve: Multiple genome alignment with gene gain, loss and rearrangement. PLoS ONE. 2010;5: e11147 10.1371/journal.pone.0011147 20593022 PMC2892488

[47] Darling ACE, Mau B, Blattner FR, Perna NT. Mauve: Multiple alignment of conserved genomic sequence with rearrangements. Genome Res. 2004;14: 1394–1403. 10.1101/gr.2289704 15231754 PMC442156

[48] O’Leary NA, Wright MW, Brister JR, Ciufo S, Haddad D, McVeigh R, et al Reference sequence (RefSeq) database at NCBI: current status, taxonomic expansion, and functional annotation. Nucleic Acids Res. 2016;44: D733–745. 10.1093/nar/gkv1189 26553804 PMC4702849

[49] Langmead B, Salzberg SL. Fast gapped-read alignment with Bowtie 2. Nat Meth. 2012;9: 357–359. 10.1038/nmeth.1923 22388286 PMC3322381

[50] Bankevich A, Nurk S, Antipov D, Gurevich AA, Dvorkin M, Kulikov AS, et al SPAdes: a new genome assembly algorithm and its applications to single-cell sequencing. J Comput Biol. 2012;19: 455–477. 10.1089/cmb.2012.0021 22506599 PMC3342519

[51] Nikolenko SI, Korobeynikov AI, Alekseyev MA. BayesHammer: Bayesian clustering for error correction in single-cell sequencing. BMC Genomics. 2013;14: S7 10.1186/1471-2164-14-S1-S7 23368723 PMC3549815

[52] Gurevich A, Saveliev V, Vyahhi N, Tesler G. QUAST: quality assessment tool for genome assemblies. Bioinformatics. 2013;29: 1072–1075. 10.1093/bioinformatics/btt086 23422339 PMC3624806

[53] Templeton AR, Crandall KA, Sing CF. A cladistic analysis of phenotypic associations with haplotypes inferred from restriction endonuclease mapping and DNA sequence data. III. Cladogram estimation. Genetics. 1992;132. 10.1093/genetics/132.2.619 PMC1205162 1385266

[54] Clement M, Posada D, Crandall KA. TCS: a computer program to estimate gene genealogies. Molecular Ecology. 2000;9: 1657–1659. 10.1046/j.1365-294x.2000.01020.x 11050560

[55] Leigh JW, Bryant D. POPART: full-feature software for haplotype network construction. Methods Ecol Evol. 2015;6: 1110–1116. 10.1111/2041-210X.12410

[56] Parks M, Cronn R, Liston A. Increasing phylogenetic resolution at low taxonomic levels using massively parallel sequencing of chloroplast genomes. BMC Biology. 2009;7: 84 10.1186/1741-7007-7-84 19954512 PMC2793254

[57] Lin C-P, Huang J-P, Wu C-S, Hsu C-Y, Chaw S-M. Comparative chloroplast genomics reveals the evolution of Pinaceae genera and subfamilies. Genome Biol Evol. 2010;2: 504–517. 10.1093/gbe/evq036 20651328 PMC2997556

[58] Pool JE, Hellmann I, Jensen JD, Nielsen R. Population genetic inference from genomic sequence variation. Genome Research. 2010;20: 291–300. 10.1101/gr.079509.108 20067940 PMC2840988

[59] Hazkani-Covo E, Zeller RM, Martin W. Molecular Poltergeists: Mitochondrial DNA Copies (numts) in Sequenced Nuclear Genomes. PLOS Genetics. 2010;6: e1000834 10.1371/journal.pgen.1000834 20168995 PMC2820518

[60] Zimmermann H, Raschke E, Epp L, Stoof-Leichsenring K, Schirrmeister L, Schwamborn G, et al The history of tree and shrub taxa on Bol’shoy Lyakhovsky Island (New Siberian Archipelago) since the last interglacial uncovered by sedimentary ancient DNA and pollen data. Genes. 2017;8: 273 10.3390/genes8100273 29027988 PMC5664123

[61] Oreshkova NV, Belokon MM, Jamiyansuren S. Genetic diversity, population structure, and differentiation of Siberian larch, Gmelin larch and Cajander larch on SSR-markers data. Genetika. 2013;49: 204–213. 10.7868/S0016675812120090 23668086

[62] Oreshkova NV, Vetrova VP, Sinelnikova NV. Genetic and phenotypic diversity of *Larix cajanderi* Mayr in the north of the Russian Far East. Contemporary Problems of Ecology. 2015;8: 9–20. 10.1134/S1995425515010096

[63] Kozyrenko MM, Artyukova EV, Reunova GD, Levina EA, Zhuravlev YN. Genetic diversity and relationships among Siberian and far eastern larches inferred from RAPD analysis. Russian Journal of Genetics. 2004;40: 401–409. 10.1023/B:RUGE.0000024978.25458.f7

[64] Wei X-X, Wang X-Q. Recolonization and radiation in Larix (Pinaceae): evidence from nuclear ribosomal DNA paralogues. Molecular Ecology. 2004;13: 3115–3123. 10.1111/j.1365-294X.2004.02299.x 15367124

[65] Semerikov VL, Lascoux M. Nuclear and cytoplasmic variation within and between Eurasian Larix (Pinaceae) species. Am J Bot. 2003;90: 1113–1123. 10.3732/ajb.90.8.1113 21659211

[66] Semerikov VL, Lascoux M. Genetic relationship among Eurasian and American *Larix* species based on allozymes. Heredity. 1999;83: 62–70. 10.1038/sj.hdy.6885310 10447704

[67] Palmer JD. Comparative organization of chloroplast genomes. Annual Review of Genetics. 1985;19: 325–354. 10.1146/annurev.ge.19.120185.001545 3936406

[68] Dong W, Xu C, Li C, Sun J, Zuo Y, Shi S, et al ycf1, the most promising plastid DNA barcode of land plants. Scientific Reports. 2015;5: 8348 10.1038/srep08348 25672218 PMC4325322

[69] Hollingsworth PM, Graham SW, Little DP. Choosing and Using a Plant DNA Barcode. PLoS One. 2011;6 10.1371/journal.pone.0019254 21637336 PMC3102656

[70] CBOL Plant Working Group, Hollingsworth PM, Forrest LL, Spouge JL, Hajibabaei M, Ratnasingham S, et al A DNA barcode for land plants. PNAS. 2009;106: 12794–12797. 10.1073/pnas.0905845106 19666622 PMC2722355

[71] Taberlet P, Coissac E, Pompanon F, Gielly L, Miquel C, Valentini A, et al Power and limitations of the chloroplast trnL (UAA) intron for plant DNA barcoding. Nucleic Acids Research. 2007;35 10.1093/nar/gkl987 17169982 PMC1807943

[72] Zimmermann HH, Raschke E, Epp LS, Stoof-Leichsenring KR, Schwamborn G, Schirrmeister L, et al Sedimentary ancient DNA and pollen reveal the composition of plant organic matter in Late Quaternary permafrost sediments of the Buor Khaya Peninsula (north-eastern Siberia). Biogeosciences. 2017;14: 575–596. 10.5194/bg-14-575-2017

[73] Sønstebø JH, Gielly L, Brysting AK, Elven R, Edwards M, Haile J, et al Using next-generation sequencing for molecular reconstruction of past Arctic vegetation and climate. Mol Ecol Resour. 2010;10: 1009–1018. 10.1111/j.1755-0998.2010.02855.x 21565110

[74] Valentini A, Taberlet P, Miaud C, Civade R, Herder J, Thomsen PF, et al Next-generation monitoring of aquatic biodiversity using environmental DNA metabarcoding. Molecular Ecology. 2016;25: 929–942. 10.1111/mec.13428 26479867

[75] Soininen EM, Zinger L, Gielly L, Bellemain E, Bråthen KA, Brochmann C, et al Shedding new light on the diet of Norwegian lemmings: DNA metabarcoding of stomach content. Polar Biology. 2013;36: 1069–1076. 10.1007/s00300-013-1328-2

[76] Willerslev E, Davison J, Moora M, Zobel M, Coissac E, Edwards ME, et al Data from: Fifty thousand years of arctic vegetation and megafaunal diet. 2014; 10.5061/dryad.ph8s5 24499916

[77] Goryachkina OV, Badaeva ED, Muratova EN, Zelenin AV. Molecular cytogenetic analysis of Siberian *Larix* species by fluorescence in situ hybridization. Plant Systematics and Evolution. 2013;299: 471–479. 10.1007/s00606-012-0737-y

[78] Kruse S, Gerdes A, Kath NJ, Epp LS, Stoof-Leichsenring KR, Pestryakova LA, et al Dispersal distances and migration rates at the arctic treeline in Siberia; a genetic and simulation based study. Biogeosciences Discussions. 2018; 1–22. 10.5194/bg-2018-267

[79] Petit RJ, Hampe A. Some evolutionary consequences of being a tree. Annual Review of Ecology, Evolution, and Systematics. 2006;37: 187–214. 10.1146/annurev.ecolsys.37.091305.110215

[80] Kruse S, Epp LS, Wieczorek M, Pestryakova LA, Stoof-Leichsenring KR, Herzschuh U. High gene flow and complex treeline dynamics of Larix Mill. stands on the Taymyr Peninsula (north-central Siberia) revealed by nuclear microsatellites. Tree Genetics & Genomes. 2018;14: 19 10.1007/s11295-018-1235-3

[81] Eisenhut G. Untersuchungen über die Morphologie und Ökologie der Pollenkörner heimischer und fremdländischer Waldbäume. P. Parey; 1961.

[82] Sjögren P, van der Knaap WO, Huusko A, van Leeuwen JFN. Pollen productivity, dispersal, and correction factors for major tree taxa in the Swiss Alps based on pollen-trap results. Review of Palaeobotany and Palynology. 2008;152: 200–210. 10.1016/j.revpalbo.2008.05.003

[83] Currat M, Ruedi M, Petit RJ, Excoffier L. The hidden side of invasions: massive introgression by local genes. Evolution. 2008;62: 1908–1920. 10.1111/j.1558-5646.2008.00413.x 18452573

[84] Du FK, Peng XL, Liu JQ, Lascoux M, Hu FS, Petit RJ. Direction and extent of organelle DNA introgression between two spruce species in the Qinghai-Tibetan Plateau. New Phytologist. 2011;192: 1024–1033. 10.1111/j.1469-8137.2011.03853.x 21883235

[85] Kuzmin DA, Feranchuk SI, Sharov VV, Cybin AN, Makolov SV, Putintseva YA, et al Stepwise large genome assembly approach: a case of Siberian larch (Larix sibirica Ledeb). BMC Bioinformatics. 2019;20: 37 10.1186/s12859-018-2570-y 30717661 PMC6362582

[86] Slatkin M, Hudson RR. Pairwise comparisons of mitochondrial DNA sequences in stable and exponentially growing populations. Genetics. 1991;129: 555–562. 1743491 10.1093/genetics/129.2.555 PMC1204643

[87] Herzschuh U, Birks HJB, Laepple T, Andreev A, Melles M, Brigham-Grette J. Glacial legacies on interglacial vegetation at the Pliocene-Pleistocene transition in NE Asia. Nature Communications. 2016;7: 11967 10.1038/ncomms11967 27338025 PMC4931021

[88] Bonan GB. Forests and Climate Change: Forcings, Feedbacks, and the Climate Benefits of Forests. Science. 2008;320: 1444–1449. 10.1126/science.1155121 18556546

[89] Andreev AA, Grosse G, Schirrmeister L, Kuzmina SA, Novenko EY, Bobrov AA, et al Late Saalian and Eemian palaeoenvironmental history of the Bol’shoy Lyakhovsky Island (Laptev Sea region, Arctic Siberia). Boreas. 2004;33: 319–348. 10.1111/j.1502-3885.2004.tb01244.x

[90] Andreev AA, Schirrmeister L, Tarasov PE, Ganopolski A, Brovkin V, Siegert C, et al Vegetation and climate history in the Laptev Sea region (Arctic Siberia) during Late Quaternary inferred from pollen records. Quaternary Science Reviews. 2011;30: 2182–2199. 10.1016/j.quascirev.2010.12.026

