# Supplementary material for: Chloroplast and mitochondrial genetic variation of larches at the Siberian tundra-taiga ecotone revealed by de novo assembly
Source: PLoS One. 2019 Jul 10;14(7):e0216966. doi: 10.1371/journal.pone.0216966 (PMC6619608; doi:10.1371/journal.pone.0216966)
Supplement: S1 Fig — The outer circle shows the positions of the 18 primer pairs for long-range PCRs. The second circle shows the re-sequenced parts of the chloroplast genomes after long-range PCR. The innermost circle represents the length of the Larix gmelinii and Larix cajanderi chloroplast genome in kilo base pairs (kb). (PDF) [file pone.0216966.s001.pdf]

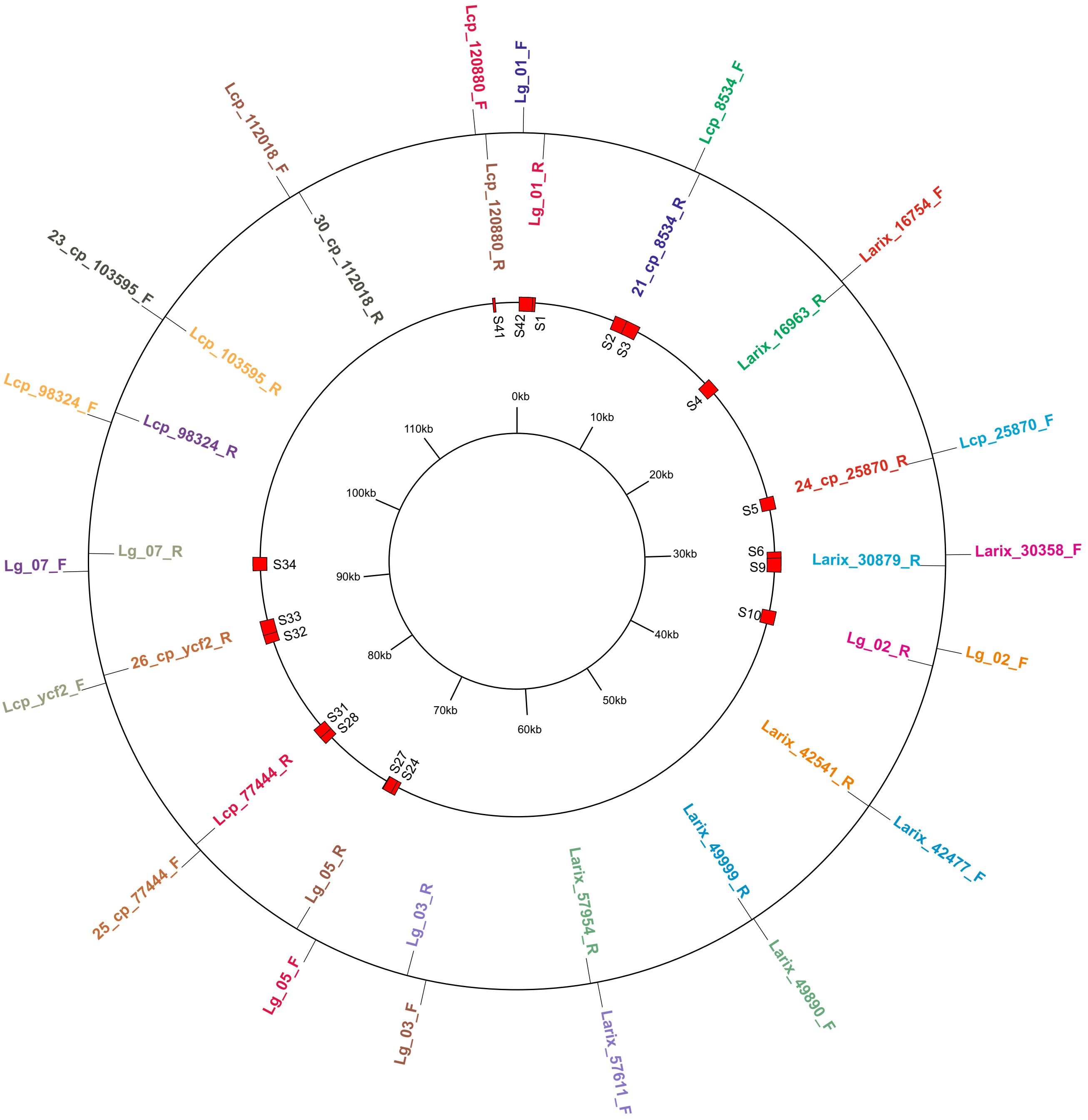

S1 Figure. Map showing the position of primer pairs for long-range PCRs. The outer circle shows the positions of the 18 primer pairs for long-range PCRs. The second circle shows the re-sequenced parts of the chloroplast genomes after long-range PCR. The innermost circle represents the length of the *Larix gmelinii* and *Larix cajanderi* chloroplast genome in kilo base pairs (kb).
